# Subacute Toxicity Profile of Lacidipine Nanoformulation in Wistar Rats

**DOI:** 10.1155/2015/947623

**Published:** 2015-05-25

**Authors:** Rupesh Shirodkar, Chandrasekhar Misra, Chethan GH, Pallavi Shetty, Zenab Attari, Srinivas Mutalik, Shaila Lewis

**Affiliations:** ^1^Department of Pharmaceutics, Manipal College of Pharmaceutical Sciences, Manipal University, Manipal, Karnataka 576104, India; ^2^Department of Pharmacy Practice, Manipal College of Pharmaceutical Sciences, Manipal University, Manipal, Karnataka 576104, India

## Abstract

The present study was aimed at investigating the safety of Lacidipine (LCDP) loaded nanostructured lipid carriers (NLCs) in Wistar rats. NLCs were formulated using ultrasound dispersion technique. Animals were orally treated once daily with NLCs containing 0.140 mg, 0.350 mg, and 0.875 mg of LCDP as low, medium, and high dose per kg body weight, respectively, during 28 days along with blank formulation and pure LCDP. Control rats were fed with water. Animals were observed throughout experiment period and their body weight was recorded once weekly. Overnight fasted rats were sacrificed on the 29th day. Study revealed no signs or symptoms of toxicity or morbidity. No significant changes in the body weight were observed between treated and control group. Significant increase in left testis weight and liver weight was observed in male and female rats, respectively. Haematological estimation revealed significant decrease in haemoglobin count in male rats while female rats showed significant increase in granulocyte count. All the serum clinical parameters were within the normal range and no gross histopathological changes were observed. No delayed effect was noted in satellite group. The results indicate that developed LCDP loaded NLCs are safe when administered orally in rats.

## 1. Introduction

Recent trend is focused on nanotechnology based drug delivery system with the aim either to improve solubility or to enhance bioavailability in order to achieve satisfactory therapeutic efficacy [1]. However, the research involving the toxicological impact and hazards of nanoparticles is still in its initial stage [2]. Among various nanoparticulate drug delivery systems, nanostructured lipid carriers (NLCs) were developed to overcome the disadvantages of solid lipid nanoparticles, namely, lower loading capacity and drug expulsion during storage [3]. Lacidipine (LCDP) was classified as per Biopharmaceutics Classification System (BCS) as class II drug [4]. LCDP is a dihydropyridine calcium channel blocker used in the treatment of hypertension with more pronounced vascular selectivity [5]. Upon oral administration, LCDP is poorly absorbed from gastrointestinal tract and undergoes extensive first pass metabolism in the liver by CYP3A4 enzymes, resulting in 2 to 9% bioavailability [6, 7]. Literature survey revealed development of NLCs for improving the bioavailability of lipophilic and low bioavailable drugs [1, 8]. To enhance the bioavailability of LCDP, it was formulated into NLCs. Earlier studies however indicated slight increase in alkaline phosphatase (ALP) level which gets normalized on long term administration [9]. LCDP was also tested in normotensive rats and results obtained from chemistry analysis revealed slight variations in the levels of creatinine kinase (CK), serum glutamate oxaloacetic transaminase (SGOT), and alanine transaminase (ALT) levels. The variations were however found to be within normal biologic limits [10]. Formulating LCDP into NLCs may improve its pharmacological activity. To evaluate whether such incorporation has any toxic effect, the subacute toxicity study of LCDP loaded NLCs formulation was carried out. Such toxicological evaluation is essential to confirm the safety of formulated nanoparticles.

## 2. Materials and Methods

### 2.1. Materials

Lacidipine, Compritol^®^ 888 ATO (Glyceryl dibehenate EP/Glyceryl behenate NF), and Lutrol^®^ F68 (Poloxamer 188) were obtained from Unichem Laboratories Ltd., Goa. Oleic acid was purchased from Sigma Aldrich, Bangalore. Ketamine injection was purchased from local pharmacy. Milli Q water used was obtained by Millipore Direct^®^ Q3, Millipore Corporation, Billerica, MA, USA. All the remaining reagents and chemicals used were of the analytical grade.

### 2.2. Preparation of LCDP Loaded NLCs and Characterization

Ultrasound dispersion technique [11] was used to formulate LCDP loaded NLCs. Compritol^®^ 888 ATO, oleic acid, and Lutrol^®^ F68 were used as solid lipid, liquid lipid, and surfactant, respectively. Briefly, solid lipid was melted at 85°C using heating mantle (5 MLH-DX, Remi Equipments Pvt. Ltd., Bengaluru) and liquid lipid was added to it. LCDP was further added to the above lipid mixture. Surfactant solution of 0.8% concentration was prepared by dissolving Lutrol^®^ F68 in Milli Q water and was added at 85°C to melted lipid phase to form coarse emulsion. Resultant emulsion was sonicated at 60 Amplitude for 8 min at pulse of 5 seconds using ultrasonic processor (VC 130, Sonics and Materials Inc., USA). The resultant nanosuspension was cooled at 4°C (ice bath) to form NLCs. The NLCs were evaluated for the particle size, polydispersity index, and zeta potential using Zetasizer nano series (Nano-ZS ZEN 3600, Malvern Instruments Ltd., UK). Entrapment efficiency was determined after extracting the LCDP in isopropyl alcohol and estimating the drug content using High Performance Liquid Chromatography (LC 2010C HT, Shimadzu Corporation, Kyoto, Japan).

### 2.3. Animal Study Protocol and Care

The study was carried out on both male and female Wistar rats aged between seven and eight weeks, supplied by Central Animal Research Facilities (CARF). Protocol was approved by Institutional Animal Ethics Committee (IAEC) of KMC Manipal vide letter IAEC/KMC/98/2012 dated 27/10/2012. Animals were kept for one week to get acclimatized to laboratory conditions. All the animals were healthy and were maintained at 22 ± 2°C and 50–60% RH in room which was well ventilated with 100% fresh air and under 12 h dark/light cycle. Animals were randomly allocated to control and treatments groups and were fed with standard pellet diet and water was provided* ad libitum* throughout the experiment period.

### 2.4. Subacute Toxicity Study

#### 2.4.1. Methodology

Repeated dose 28-day oral toxicity study was performed as per the Organization of Economic Cooperation and Development (OECD) guideline 407 for testing of chemicals [12]. In the present study, seventy Wistar rats (35 males and 35 females) weighing 150 ± 50 g were divided into seven groups and each group had 10 rats (5 rats of each sex). Group 1 served as control and received Milli Q water at dose of 10 mL/kg body weight. Group 2 received blank formulation (formulation without drug) at dose of 10 mL/kg body weight. Group 3 received LCDP drug dispersion prepared in 0.25% CMC at dose of 0.350 mg/kg body weight. Groups 4, 5, and 6 received the NLCs containing 0.140 mg, 0.350 mg, and 0.875 mg of LCDP as low, medium, and high dose/kg body weight, respectively. Group 7 served as satellite group which received NLCs containing 0.875 mg of LCDP/kg body weight for 28 days to determine reversibility or recovery from the toxic effect. Satellite group was then observed for the next 14 days without LCDP loaded NLCs administration.

#### 2.4.2. Observation

During the study period (before and after dose), all the animals were observed twice daily for mortality and morbidity. Daily intake of food and water consumption was monitored. Once daily clinical observation was made following the administration of control, blank formulation, standard drug, and nanoformulation to detect sign of toxicity like general behavior, motor activities, reflexes, and changes in skin and fur texture. Body weight of animal was recorded once a week [13].

#### 2.4.3. Blood Analysis

After completion of treatment period, overnight fasted (ad libitum drinking water) rats were sacrificed under ketamine anesthesia. Before sacrifice, blood samples were collected by retro orbital plexus puncture in tubes containing anticoagulant dipotassium ethylene diamine tetra acetic acid (K_2_ EDTA) for the estimation of haematological parameters using Veterinary Blood Cell Counter (PCE-21OVET, ERMA, Inc., Tokyo). Blood samples were also collected in tubes and serum was separated by centrifugation (3 K 30 Sigma Laborzentrifugen GmbH, Germany) at 4°C at 7000 rpm for 5 min and stored at −20°C until estimation of clinical parameters was carried out. Clinical parameters such as ALT, SGOT, ALP, creatinine (CRE), uric acid (UA), total protein (TP), CK, and bilirubin (BIL) were analysed using Roche 111 autoanalyser with Cobas kits.

#### 2.4.4. Histopathology

After sacrifice and blood collection, brain, liver, heart, lungs, kidneys, testis, spleen, ovaries, and stomach were collected from the animals and placed in 0.9% ice cold sodium chloride solution for 30 min before recording of their weights. Subsequently, brain, heart, kidney, and liver of rats fed with high dose NLC formulation, control, and blank formulation were stored in buffered formalin solution until histopathological examination was performed. Sections of 5 *µ*m thickness were cut, stained with haematoxylin and eosin, and examined under the light microscope with high power magnification. All the examinations were done by qualified pathologist.

#### 2.4.5. Satellite Group

Satellite group animals were observed daily for 14 days after 28-day dosing to check for any withdrawal symptoms associated during recovery period. On the 42nd day, animals were sacrificed and major organs were visually inspected for any abnormalities.

#### 2.4.6. Statistical Analysis

Results were expressed as mean ± SEM. Analysis of data for change in body weight was performed by two-way analysis of variance (ANOVA) using Bonferroni* post hoc* test. All other data were subjected to one-way ANOVA with Dunnett's* post hoc* test to evaluate significant differences between the groups using GraphPad Prism 5.0 software. Results were considered to be significant at *p* < 0.05.

## 3. Results

### 3.1. Characterization of NLC Formulation

The optimized LCDP loaded NLCs formulation was evaluated for particle size, polydispersity index, zeta potential, and entrapment efficiency. The results are shown in Table 1.

### 3.2. Subacute Toxicity Results

#### 3.2.1. General Sign in the Rat

All the animals fed with the treatment group were found to be healthy. There were no changes observed in their behavior and locomotor activity. There was absence of any visual sign of intoxication during the 28-day period. There were no changes observed in morphological characteristics of skin, eyes, nose, and fur. Animals also showed normal nutritional status.

#### 3.2.2. Body Weight

**Figure 1:**
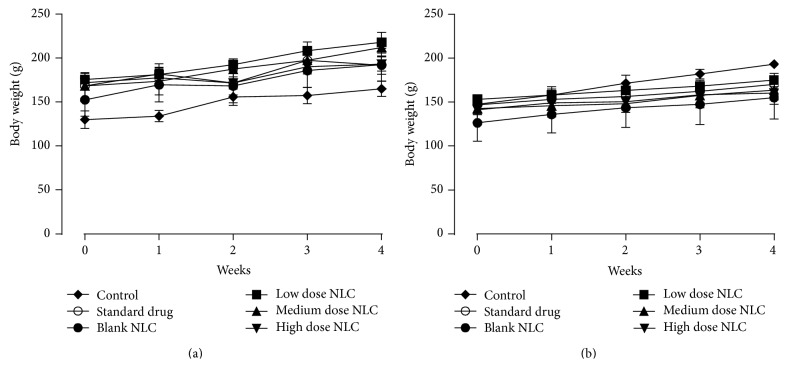
Change in body weight of (a) male rats and (b) female rats treated with NLCs formulation. All the values are represented as mean ± SEM and *n* = 5.

Nonsignificant (*p* < 0.05) increase in body weight was observed in all the groups. Decrease in body weight gain is one of the indicators of adverse effect [14]. Daily food and water consumption was found to be normal in both control and treatment groups. Hence, we can assume that developed NLCs of LCDP do not affect the normal growth of rats [15]. Graphical representation of changes in body weight is shown in Figure 1.

#### 3.2.3. Organ Weight

**Figure 2:**
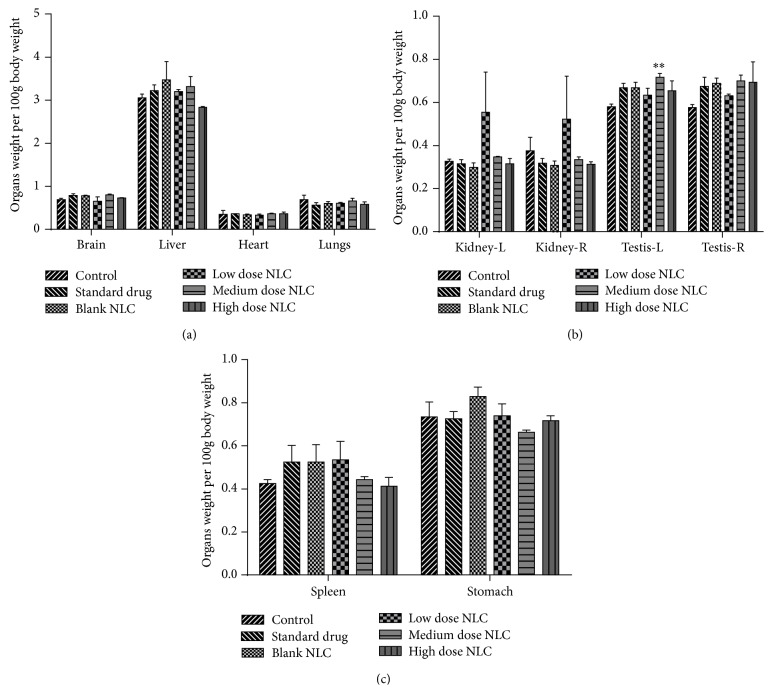
(a, b, and c) Organ weight of male rats treated with NLCs formulation. All the values are represented as mean ± SEM and *n* = 5. ^*∗∗*^ *p* < 0.01. Kidney-R: right kidney, Kidney-L: left kidney, Testis-R: right testis, and Testis-L: left testis.

**Figure 3:**
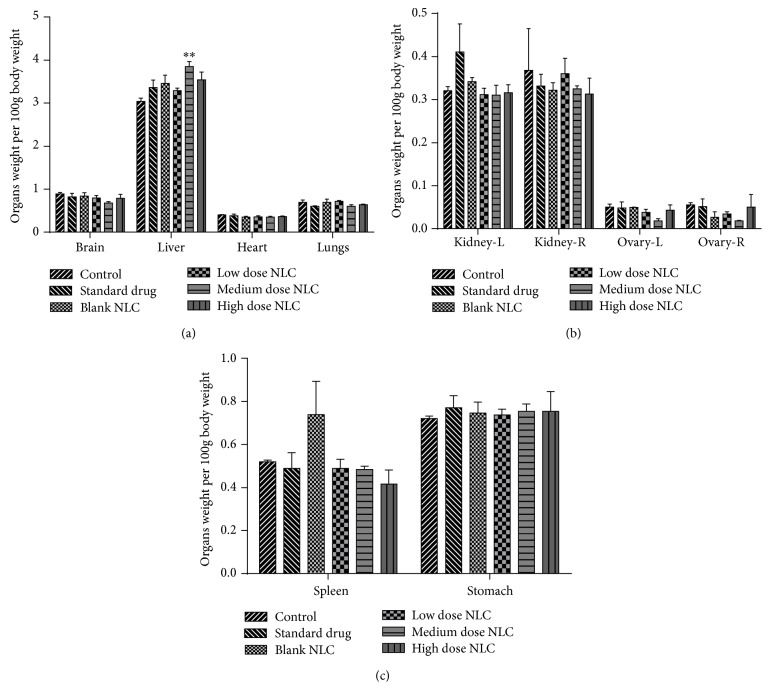
(a, b, and c) Organ weight of female rats treated with NLCs formulation. All the values are represented as mean ± SEM and *n* = 5. ^*∗∗*^ *p* < 0.01. Kidney-R: right kidney, Kidney-L: left kidney, Ovary-R: right ovary, and Ovary-L: left ovary.

Organ weight per 100 g body weight was determined for different organs. Significant increase (*p* < 0.01) only in left testis weight and liver weight was observed in medium dose treated male and female rats, respectively. Weight of other organs did not change significantly. Graphical representation of organ weight is shown in Figures 2 and 3.

#### 3.2.4. Hematological Parameters

**Figure 4:**
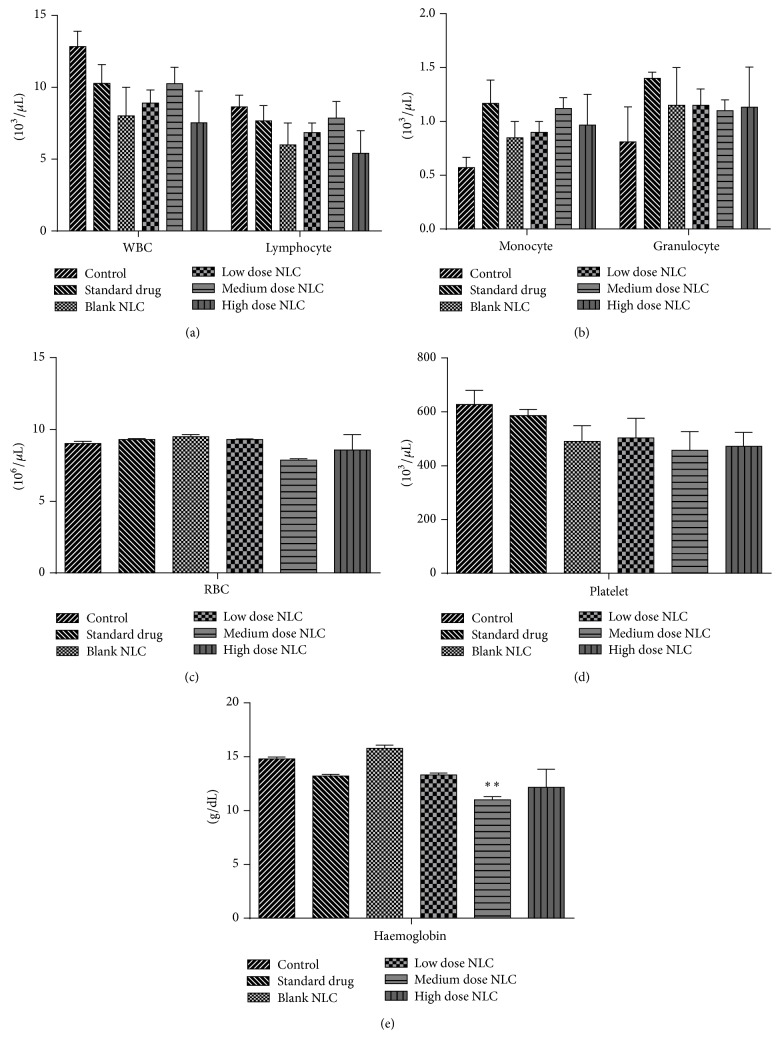
(a, b, c, d, and e) Hematological count of male rats treated with NLCs formulation. All the values are represented as mean ± SEM and *n* = 5. ^*∗∗*^ *p* < 0.01.

**Figure 5:**
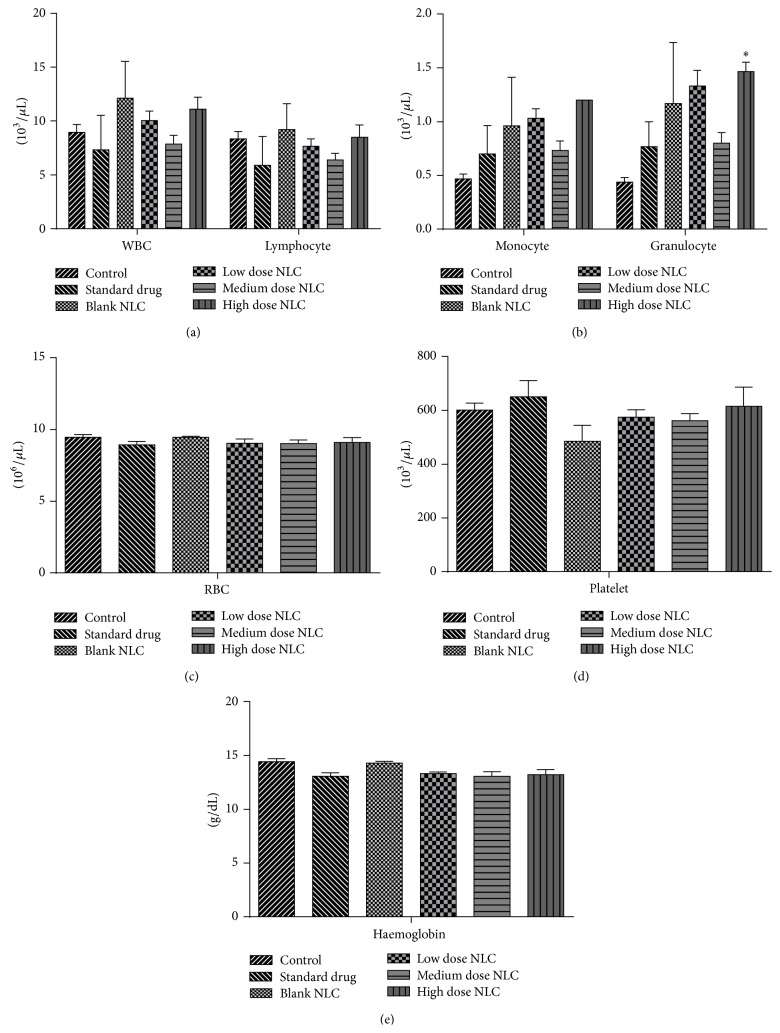
(a, b, c, d, and e) Hematological count of female rats treated with NLCs formulation. All the values are represented as mean ± SEM and *n* = 5. ^*∗*^ *p* < 0.05.

When compared with the control, haematological estimation revealed only significant decrease (*p* < 0.01) in haemoglobin count in medium dose treated male rats. There were no significant changes in RBC, WBC, granulocytes, lymphocytes, monocyte, and platelet counts in treated male rats (Figure 4). Female rats showed only significant increase (*p* < 0.05) in granulocyte count in high dose treated rats. Counts of RBC, WBC, lymphocytes, platelet, monocytes, and haemoglobin remained unchanged in the treated female rats (Figure 5).

#### 3.2.5. Serum Clinical Chemistry

**Figure 6:**
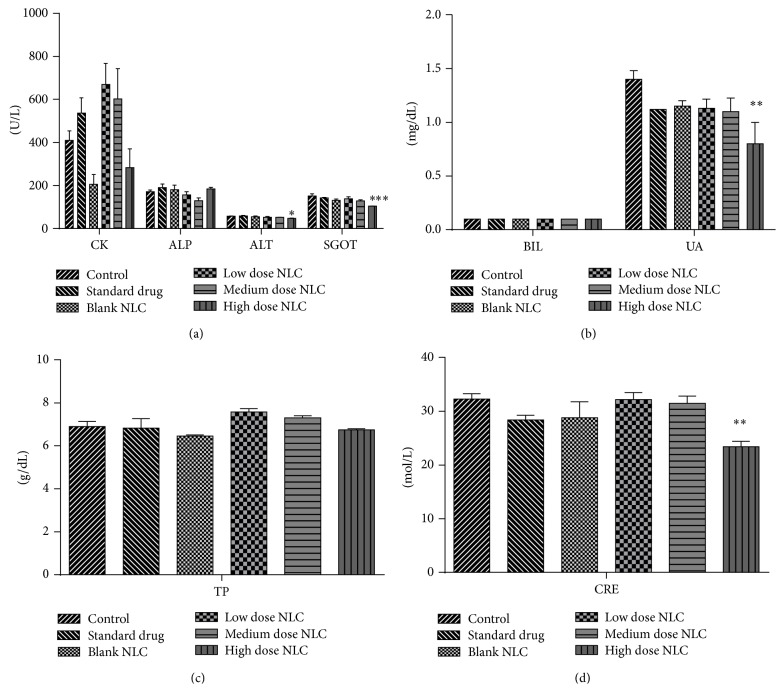
(a, b, c, and d) Clinical parameters of male rats treated with NLCs formulation. All the values are represented as mean ± SEM and *n* = 5. ^*∗*^ *p* < 0.05, ^*∗∗*^ *p* < 0.01, and ^*∗∗∗*^ *p* < 0.001.

**Figure 7:**
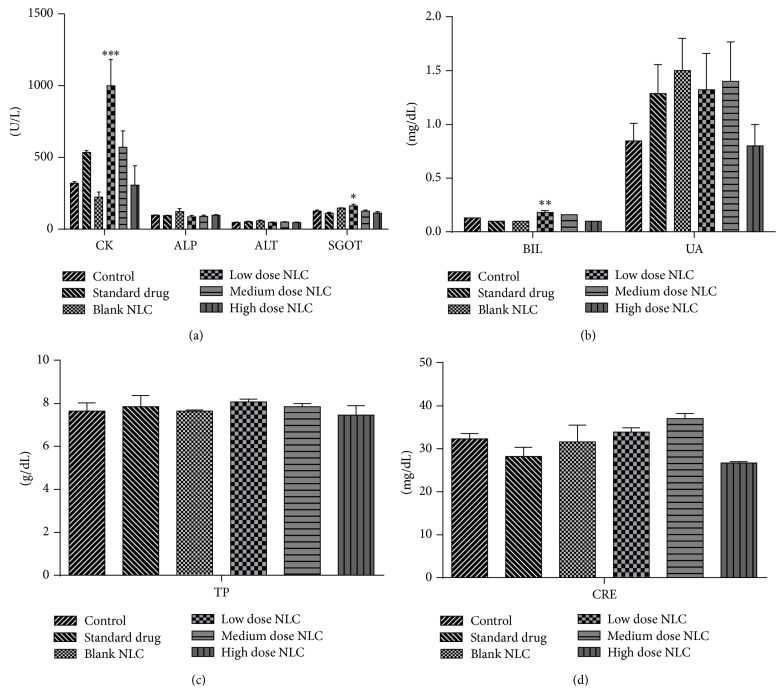
(a, b, c, and d) Clinical parameters of female rats treated with NLCs formulation. All the values are represented as mean ± SEM and *n* = 5. ^*∗*^ *p* < 0.05, ^*∗∗*^ *p* < 0.01, and ^*∗∗∗*^ *p* < 0.001.

Clinical investigation revealed significant decrease in the levels of ALT (*p* < 0.05), SGOT (*p* < 0.001), CRE (*p* < 0.01), and UA (*p* < 0.01) in high dose treated male rats. Levels of ALP, BIL, CK, and TP did not change significantly (Figure 6). Female rats showed significant increase in the levels of CK (*p* < 0.001), SGOT (*p* < 0.05), and BIL (*p* < 0.01) in low dose treated formulation. No significant difference was observed in the levels of ALP, ALT, CRE, UA, and TP in female rats (Figure 7).

#### 3.2.6. Histopathological Examination

**Figure 8:**
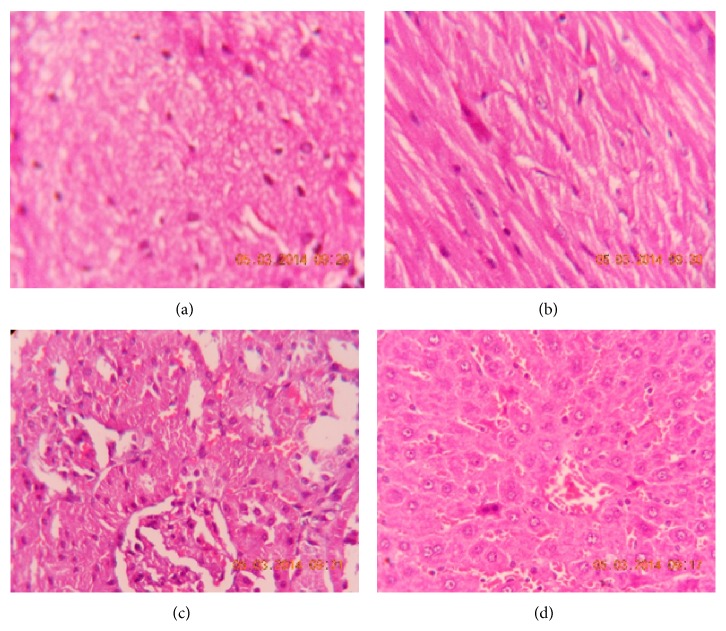
Photomicrograph of (a) brain, (b) heart, (c) kidney, and (d) liver of control group.

**Figure 9:**
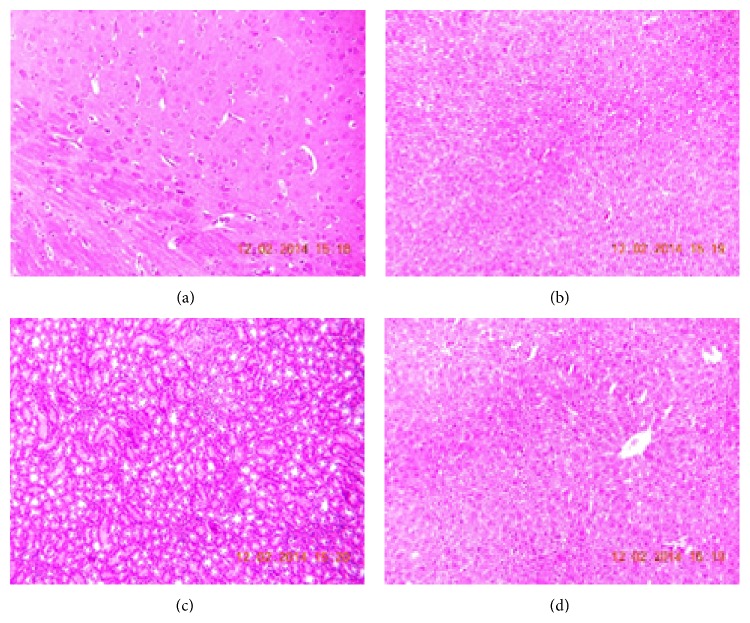
Photomicrograph of (a) brain, (b) heart, (c) kidney, and (d) liver of group treated with blank formulation.

**Figure 10:**
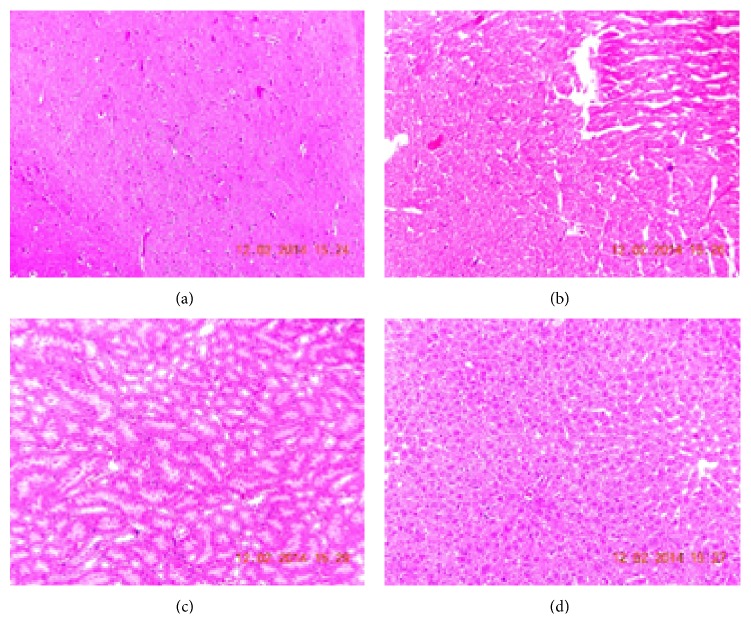
Photomicrograph of (a) brain, (b) heart, (c) kidney, and (d) liver of group treated with NLCs formulation.

Histopathological examination of brain, heart, kidney, and liver did not reveal any changes in the rats fed with control, blank formulation, and LCDP loaded NLCs formulation (Figures 8, 9, and 10). Normal architecture was observed in the sections of organs from all the groups. Detrimental changes were not observed. There were no signs of any morphological disorders like inflammation, fibrosis, necrosis, and steatosis induced by oral administration of blank formulation and LCDP loaded NLCs for 28 days when compared with control thereby confirming the safety of LCDP nanoformulation.

#### 3.2.7. Satellite Group Examination

Animals from satellite group were observed daily for the next 14 days after 28 days of daily dosing. Animals did not show any sign of withdrawal symptoms. Animals were sacrificed and organs were inspected and found to possess normal architecture.

## 4. Discussion

Repeated dose 28-day oral toxicity study was performed on developed NLCs of LCDP using Wistar rats. Nanoformulation of LCDP was formulated to increase its bioavailability. Variation in the toxicity results was seen in both male and female rats. No mortality or morbidity was observed in any of male and female rats after oral administration of the nanoformulation of LCDP for the 28-day study period. There was no sign of any clinical abnormalities in any of the treated male and female rats. Organ weight monitoring is important parameter in toxicological studies [16]. Reduction in body weight gain and the weights of the internal organs is index of toxicity after exposure to toxic compounds [17, 18]. Compared with the control group, the body weight gain of treated male and female rats was not significantly different suggesting no adverse effect on the body weight. When compared with control, there was significant increase of 18% and 21% in the left testis and liver weight in medium dose treated male and female rats, respectively. Weight of other organs did not change significantly in the treated rats. Although weight of left testis and liver is significantly different when compared with control, no gross morphological changes were observed suggesting that developed formulation is virtually nontoxic. Drug is metabolized by the liver and increase in its weight may be due to response to compensate for increase in demand of its metabolism [19]. Haematological parameters remained unaltered between control and treatment group after 28 days of treatment except for decrease in haemoglobin count in medium dose treated male rats and increase in granulocyte count in high dose treated female rats. Haematopoietic system is most sensitive target of toxic compounds which defines the physiological and pathological status in men and animals [20, 21]. Changes only in haemoglobin and granulocyte count do not indicate hematotoxicity. All the values were within the normal limits and hence the result is considered normal for this species [22]. This indicates that the developed nanoformulation is nontoxic to the blood cell. Levels of serum marker enzymes are used in routine clinical evaluation of health status [23]. Levels of ALT and SGOT are largely used in the assessment of liver damage by drug or hepatotoxins [24]. Elevation of the levels of ALT and SGOT is an indicator of liver and heart damage [25, 26]. Biochemical investigation revealed decrease in the levels of ALT, SGOT, CRE, and UA in high dose treated male rats. The significant decrease in the levels of ALT and SGOT indicates that the formulation may not produce liver toxicity and will not produce toxic effect on heart tissue. There were no significant increases in CRE and UA levels suggesting no renal impairment or kidney damage [27]. Since the levels were decreased as compared to control, the effect may be nonsignificant clinically. Levels of other clinical parameters were unaltered. Significant increases only in the levels of CK, SGOT, and BIL were observed in low dose treated female rats. Medium dose and high dose treated female rats did not show significant difference which indicates that difference observed in levels of CK, SGOT, and BIL in low dose treated rats may be due to variation in the animals. LCDP is excreted by biliary route and histopathological investigation did not reveal any alteration in the liver tissue. Normal liver function was also confirmed by the levels of TP [10]. No sign of impaired renal function was also indicated by the fact that there was no significant increase in the levels of TP [28]. No significance difference in the levels of other clinical parameters between control and treated group was observed in female rats. Although there was variation in the levels of few serum clinical parameters in both treated male and female rats, histopathological investigation of major organs did not reveal any alteration or pathological condition which confirms the safety of developed nanoformulation.

## 5. Conclusions

LCDP loaded NLCs were formulated to increase its bioavailability and subacute toxicity study of developed nanoformulation was carried out. The results confirm the safety of developed LCDP loaded NLCs formulation and were found to be relatively free from toxicity when administered orally in rats.

## Figures and Tables

**Table 1:** Results of optimized LCDP loaded NLC formulation.

Parameters	Results
Particle size (nm)	88.89 ± 5.020
Polydispersity index	0.221 ± 0.023
Zeta potential (mV)	−30.7 ± 6.83
Entrapment efficiency (%)	74.1 ± 0.105

Data is presented as mean ± SEM and *n* = 3.
